# Depression symptoms and quality of life in empty-nest elderly among Chengdu: A cross-sectional study

**DOI:** 10.3389/fpsyt.2022.1003261

**Published:** 2022-11-08

**Authors:** Lanying He, Jian Wang, Feng Wang, Lili Zhang, Yinglin Liu, Fan Xu

**Affiliations:** ^1^Department of Neurology, The Second People's Hospital of Chengdu, Chengdu, China; ^2^Department of Public Health, Chengdu Medical College, Chengdu, China

**Keywords:** empty-nest elderly, quality of life, depression, mental health, WHOQOL-BREF

## Abstract

**Objectives:**

To estimate the prevalence of depression symptoms and quality of life (QoL) and examined the influence of factors in the empty nest elderly.

**Methods:**

This was a cross-sectional study, which was conducted from February 2022 to May 2022. We recruited a convenience sample of no empty-nest elderly and empty-nest elderly (≥60 years) living in Chengdu. QoL was assessed using WHOQOL-BREF, Geriatric Depression Scale (GDS-15) was used to assess depression symptoms. Multivariable logistic regression was used to analyze data between independent variables with depression symptoms.

**Results:**

Two thousand twenty-six participants were included in this study, 39.0% (660/1,082) experienced depression symptoms among empty-nest elderly. Age (aOR, 1.02; 95% CI, 1.00–1.04; *P* = 0.046), chronic disease≥2 (aOR, 3.29; 95% CI, 2.50–4.33; *P* < 0.001) were associated with increased risk of depression symptoms, and physical activity (aOR, 0.59; 95% CI, 0.40–0.87; *P* = 0.008), physical health (aOR, 0.93; 95% CI, 0.88–0.99; *P* = 0.026), psychological health (aOR, 0.93; 95% CI, 0.87–0.98; *P* = 0.013), and total score (aOR, 0.97; 95% CI, 0.96–0.99; *P* < 0.001) were associated with decreased risk of depression symptoms among empty-nest elderly.

**Conclusions:**

Depression symptoms are common mental health problems among empty-nest elderly. We found that age, chronic disease ≥2 and physical activity were important factors that have an impact on depressive symptoms. Empty-nest elderly would have lowered QoL score.

## Introduction

With the improvement of social economy and health care, the older population (aged ≥60 years) now comprises the fastest-growing age group worldwide, which is a major social problem facing the world in the 21st century. According to the seventh demographic census, the elderly aged 60 and above account for 264 million people in China, accounting for 18.7% of the total population (1). According to the 2016 China Family Development Report released by the National Health and Family Planning Commission, empty-nest elderly account for about half of the elderly population in China, and the proportion is likely to reach more than 90% by 2030 (2). With the increasing number of empty-nest elderly, the physical and psychological problems of empty-nest elderly have become an urgent social problem.

Empty-nest elderly are more likely to experience reduced physical function, psychological stress, and social problems, and may also have a worse quality of life (QoL) due to worse health associated with aging and inadequate social support (3, 4). Elderly generally have a higher risk of mental illness than younger adults, such as anxiety, depressive symptoms and loneliness, which may have negative effects on physical health, cognition, and even lead to self-injury and suicidal behavior (5, 6). Many studies found that empty-nest elderly had a significantly higher prevalence of depression than those living with children (7–9), and also found some risk factors those associated with depression symptoms (10–12), however, due to different variable included, the results of these studies have been inconsistent. These previous studies have been conducted for a long time, which cannot represent the current situation, and cannot represent the current situation of southwest China. So far, few studies have investigated the relationship between depression symptoms and QoL among empty-nest elderly.

Because of the one-child policy implemented in the past 30 years, most Chinese families have only one child, children grow up and leave the family, their parents become empty-nest elderly. Chengdu is a central city in southwest area, has above 15 million people, ranking ninth in China, and about 20 % people is over 60, and investigating the prevalence of depression symptoms and related risk factors in empty-nest elderly is important for developing preventive measures and allocating health resources to reduce the negative impact of depressive symptoms on health and QoL.

Therefore, the aim of the present study was to estimate the prevalence of depression symptoms and quality of life (QoL) and examined the influence of factors in the empty nest elderly among the Chengdu.

## Methods

This was a cross-sectional study, which was conducted from February 2022 to May 2022. We selected 15 districts/counties in Chengdu as the study sites. First, the streets/townships of each district/county are numbered according to the district/county order on the government website. Second, the random number table method is used to select 2 communities/administrative villages in each street/township, and scientifically select random samples. Third, two residential areas/villages were extracted from each community/administrative village.

The inclusion criteria were: (1) Aged 60 years and above; (2) Living in the survey site for more than 1 year; (3) Living at home; (4) Consent to participate in the study. The exclusion criteria were: (1) Unable to complete the questionnaire, such as deafness, deafness, and other severe disease; (2) End-stage Alzheimer's disease and Parkinson's disease; (3) End-stage heart disease; (4) Other serious psychiatric disorders besides depression.

All data were collected online, and assessment of the scale was performed by trained neurologists. The collected data can be automatically entered into a spreadsheet. The demographic variables included gender, age, education level, smoking, alcohol, physical activity, chronic disease, household income (yes/no). The researchers were accompanied by a community physician, visited each eligible participant and explained the purpose and process of the study. Each participant was invited to answer questionnaires after providing written informed consent. The answers are then checked by quality control personnel to ensure completeness, accuracy and consistency. The participants took 25 to 30 min completing the scale and questionnaire.

Chronic diseases included cancer, hypertension, diabetes, mild heart disease, chronic obstructive pulmonary disease (COPD), liver dysfunction, kidney dysfunction, asthma, stroke, epilepsy, multiple sclerosis, mild Alzheimer's disease, and mild Parkinson's disease.

Physical activity was defined as exercise ≥30 min at least 3 times a week regularly during the past year, included: walking, hiking, biking, aerobics, swimming, and other activities. Empty-nest elderly were defined as those who did not live with their children more than 1 year, they lived with their spouse or/with a caregiver or alone. The data was obtained by the question: “Do you live with your children in recent 1year?”

The World Health Organization Quality of Life-brief version (WHOQOL-BREF) was used to assess QoL. WHOQOL-BREF is a short version of the original WHOQOL-100 developed by WHO (13), which is a self-reported questionnaire, and contains a total of 26 questions, the first two of which are about their overall QoL and their general perception of their health. The remaining 24 questions covered four areas: 7 physical health questions, 6 mental health questions, 3 social relationship questions and 8 environmental questions, each on a scale of five to five. Each question is 5 score on a scale of 1 to 5. The domain score is calculated by averaging the score for each entry within the domain, which is then multiplied by four to maintain comparability with the score used in WHOQOL-100. The Chinese version of WHOQOL-BREF used in this study has good internal consistency and Cronbach's α coefficient is 0.95 (14).

Depression symptoms were detected using the Geriatric Depression Scale-15 (GDS-15), which was developed to measure depression symptoms in the elderly (15). Higher scores indicate more severe depression symptoms. Depression symptoms were defined as a score of five or more. GDS-15 ≥ 5 has the best sensitivity and specificity for the diagnosis of depression symptoms (16).

All participants were informed of the study procedures at the time of recruitment. After signing informed consent, participants were interviewed by a trained neurologist. If the participant is in poor health, written consent must be signed by his/her legal guardian. A total of 2,071 participants were invited to answer a standardized questionnaire, and 2,026 completed the study. This study was approved by the Ethics Committee of the second people's hospital of Chengdu.

### Statistical analysis

The data were presented as numbers (%), or mean values (±standard deviation, SD). To identify differences between two groups, the Pearson χ^2^ test was used for categorical variables. Student's *t*-test was used to compare normally distributed variables. Mann-Whitney U test was used to compare non-normally distributed variables. Variables associated with depression symptoms in the univariate analyses with a *P*-value < 0.20 were included in the multivariate analysis. Multivariate logistic regression analysis was performed to identify risk factors associated with depression symptoms. The results were expressed as adjusted odds ratios (aOR) with their corresponding 95% confidence intervals (CI). The data were analyzed using SPSS 22 software. *P* < 0.05 was considered statistically significant.

## Results

### Demographic characteristic

Two thousand twenty-six participants were included (mean age was 69.11 years), 53.4% were female, 70.83% had received middle school education, 9.9% had smoked and 30.4% drunk alcohol. A total of 57.00% of the participants had two or more kinds of chronic diseases. Only 15.7% of the participants had physical activity. The GDS-15 score was 4.34 ± 2.48, 609 (30.06%) participants had GDS-15 ≥ 5 (Table 1).

**Table 1:** Comparison of baseline characteristics between empty-nest elderly and no empty-nest elderly groups.

	**Empty-nest**	**No empty-nest**	**OR (95% CI)**	P^*^
	**elderly (1,082)**	**elderly (944)**		
Age, y (Mean SD)	69.30 ± 7.08	68.87 ± 6.73		0.200
GDS-15, (Mean SD)	4.76 ± 2.57	3.86 ± 2.29		**<0.001**
GDS-15 ≥ 5, *n* (%)	422 (39.0)	187 (19.8)	2.59 (2.12–3.17)	**<0.001**
Females, *n* (%)	564 (52.1)	518 (54.9)	0.89 (0.75–1.07)	**0.244**
**Education**				
Elementary school, *n* (%)	140 (12.9)	145 (15.4)	0.82 (0.64–1.05)	0.133
Middle school, *n* (%)	766 (70.8)	669 (70.9)	1.00 (0.82–1.21)	0.971
High school and above, *n* (%)	176 (16.3)	130 (13.8)	1.22 (0.95–1.56)	0.118
Smoking, *n* (%)	106 (10.1)	95 (9.8)	0.97 (0.73–1.30)	0.841
Alcohol, *n* (%)	329 (30.4)	287 (30.4)	1.00 (0.83–1.21)	0.998
Physical activity, *n* (%)	166 (15.3)	153 (16.2)	0.94 (0.74–1.19)	0.594
Chronic disease ≥ 2, *n* (%)	618 (57.1)	537 (56.9)	1.01 (0.85–1.20)	0.917
**Household income, Chinese Yuan**				
Low household income (≤5,000 CNY/month), *n* (%)	228 (21.1)	230 (24.4)	0.83 (0.67–1.02)	0.077
Middle household income (5,000–8,000CNY/month), *n* (%)	581 (53.7)	496 (52.5)	1.05 (0.88–1.25)	0.603
High household income (>8,000 CNY/month), *n* (%)	273 (25.2)	218 (23.1)	1.12 (0.92–1.38)	0.263
**Quality of life**				
Physical health, (Mean SD)	13.99 ± 2.15	14.70 ± 2.01		**<0.001**
Psychological health, (Mean SD)	13.44 ± 2.18	13.86 ± 2.34		**<0.001**
Social relationships, (Mean SD)	15.48 ± 2.78	15.32 ± 2.82		0.268
Environment, (Mean SD)	14.07 ± 2.65	14.25 ± 2.59		0.256
Total score, (Mean SD)	81.59 ± 9.90	84.49 ± 8.72		**<0.001**

Bold indicates P-values < 0.05.

* Comparison between empty-nest elderly and no empty-nest elderly groups. The data are presented as numbers (%) or mean values (±standard deviation). The Pearson χ^2^ test was used for categorical variables. Student's t-test was used to compare normally distributed variables. Mann-Whitney U tests were used to compare non-normally distributed variables.

One thousand eighty-two participants were empty-nest elderly, the mean age was 69.30 ± 7.08 years (60–89 Years), GDS-15 score were 4.76 ± 2.57, among them, 39.0% had GDS-15 ≥ 5. There were significant differences in GDS value, the percentage of GDS-15 ≥ 5, physical activity, physical health, psychological health, total score among the two groups (empty-nest elderly, no empty-nest elderly), while there were no significant differences in chronic disease ≥ 2, education level, household income among the two groups. Empty-nest elderly had the higher GDS-15 scores, higher percentage of GDS-15 ≥ 5, lower physical activity, physical health, psychological health, total score (Table 1).

### The risk factors for depression symptoms among all participants

Of the 2,026 participants, 609 (30.1%) participants had GDS-15 ≥ 5. The average GDS-15 of participants with depression symptoms was 7.43 ± 1.54 and the average GDS-15 score of participants with no depression symptoms was 3.01 ± 1.39. Baseline characteristics of participants in the no depression and depression symptoms groups were compared (Table 2). The results showed that participants with GDS-15 ≥ 5 had higher the percentage of empty-nest elderly and chronic disease ≥ 2, lower physical activity, and lower physical health, psychological health, total score (*P* < 0.05).

**Table 2:** Comparison of baseline characteristics between no depression and depression symptoms groups.

	**No depression (1,417)**	**Depression (609)**	**OR (95% CI)**	P^*^
Age, y (Mean SD)	68.84 ± 6.75	69.76 ± 7.28		**0.006**
GDS-15, (Mean SD)	3.01 ± 1.39	7.43 ± 1.54		**<0.001**
Empty-nest, *n* (%)	660 (46.6)	422 (69.3)	2.59 (2.12–3.17)	**<0.001**
Females, *n* (%)	749 (52.9)	333 (54.7)	1.08 (0.89–1.30)	0.451
Education (MSc and above education), *n* (%)	1,159 (85.72)	146 (86.39)	1.06 (0.66–1.68)	0.815
Elementary school, *n* (%)	187 (13.2)	98 (16.1)	1.26 (0.97–1.65)	0.086
Middle school, *n* (%)	1,023 (72.2)	412 (67.7)	0.82 (0.66–0.99)	**0.039**
High school and above, *n* (%)	207 (14.6)	99 (16.3)	1.14 (0.87–1.47)	0.342
Smoking, *n* (%)	144 (10.2)	57 (9.4)	0.91 (0.66–1.26)	0.579
Alcohol, *n* (%)	419 (29.6)	197 (32.3)	1.14 (0.93–1.40)	0.213
Physical activity, *n* (%)	246 (17.4)	73 (12.0)	0.70 (0.53–0.93)	**0.012**
Chronic disease ≥ 2, *n* (%)	735 (51.9)	420 (69.0)	2.06 (1.69–2.52)	**<0.001**
**Household income, Chinese Yuan**				
Low household income (≤5,000 CNY/month), *n* (%)	309 (21.8)	149 (24.5)	1.16 (0.93–1.45)	0.189
Middle household income (5,000–8,000 CNY/month), *n* (%)	452 (53.1)	325 (53.4)	1.01 (0.84–1.22)	0.902
High household income (>8,000 CNY/month), *n* (%)	356 (25.1)	135 (22.2)	0.85 (0.68–1.06)	0.155
**Quality of life**				
Physical health, (Mean SD)	14.46 ± 2.06	14.00 ± 2.20		**<0.001**
Psychological health, (Mean SD)	13.74 ± 2.31	13.38 ± 2.13		**<0.001**
Social relationships, (Mean SD)	15.43 ± 2.80	15.37 ± 2.81		0.438
Environment, (Mean SD)	14.12 ± 2.64	14.24 ± 2.56		0.319
Total score, (Mean SD)	83.68 ± 9.02	81.22 ± 810.30		**<0.001**

Bold indicates P-values < 0.05.

* Comparison between no depression and depression symptoms groups. The data are presented as numbers (%) or mean values (±standard deviation). The Pearson χ^2^ test was used for categorical variables. Student's t-test was used to compare normally distributed variables. Mann-Whitney U tests were used to compare non-normally distributed variables.

### Multivariable models on the association between GDS-15 ≥ 5 and risk factors among all participants

In the all participants, to further analyze the risk factors for the GDS-15 ≥ 5, variables associated with depression symptoms in the univariate analyses with a *P*-value < 0.20 were included in the multivariate analysis, after adjusting for age, empty-nest, elementary school, middle school, physical activity, chronic disease ≥ 2, low household income, high household income, physical health, psychological health, total score, the results shown that age (aOR, 1.02; 95% CI, 1.00–1.03; *P* = 0.040), empty-nest (aOR, 2.38; 95% CI, 1.92–2.93; *P* < 0.001) were associated with increased risk of depression symptoms (Table 3), and physical activity, physical health, psychological health, total score were associated with decreased risk of depression symptoms (Table 3).

**Table 3:** Multivariable models showing factors associated with GDS-15 ≥ 5 among all participants.

	**OR (95% CI)**	P^*^
Age, y (Mean SD)	1.02 (1.00–1.03)	**0.040**
Empty-nest	2.38 (1.92–2.93)	**<0.001**
Physical activity	0.70 (0.53–0.93)	**0.012**
Chronic disease ≥ 2	2.06 (1.69–2.52)	**<0.001**
Physical health	0.94 (0.90–0.99)	**0.016**
Psychological health	0.95 (0.91–1.00)	**0.041**
Total score	0.98 (0.97–0.99)	**<0.001**

Bold indicates P-values < 0.05.

* Multivariable adjusted for age, empty-nest, elementary school, middle school, physical activity, chronic disease ≥ 2, low household income, high household income, physical health, psychological health, total score.

### The risk factors for depression symptoms among empty-nest elderly

In the empty-nest elderly, 660 participants had GDS-15 ≥ 5. Among GDS-15 ≥ 5 group, there was higher percentage of chronic disease ≥ 2, and lower percentage of physical activity, physical health, psychological health, total score in GDS-15 ≥ 5 group were significantly lower than that in GDS-15 < 5 group (Table 4).

**Table 4:** Baseline characteristics between no depression and depression symptoms among empty-nest elderly.

	**No depression (422)**	**Depression (660)**	**OR (95% CI)**	P^*^
Age, y (Mean SD)	68.82 ± 6.96	70.06 ± 7.21		**0.004**
GDS−15, (Mean SD)	3.07 ± 1.40	7.41 ± 1.55		**<0.001**
Females, *n* (%)	322 (50.3)	232 (55.0)	1.21 (0.94–1.54)	0.133
Education (MSc and above education), *n* (%)	1,159 (85.72)	146 (86.39)	1.06 (0.66–1.68)	0.815
Elementary school, *n* (%)	75 (11.4)	65 (15.4)	1.42 (0.99–2.03)	0.054
Middle school, *n* (%)	480 (72.7)	286 (67.8)	0.79 (0.60–1.03)	0.080
High school and above, *n* (%)	105 (14.6)	71 (16.3)	1.07 (0.77–1.49)	0.691
Smoking, *n* (%)	71 (10.8)	35 (8.3)	0.75 (0.49–1.15)	0.184
Alcohol, *n* (%)	195 (29.5)	134 (31.8)	1.11 (0.85–1.45)	0.441
Physical activity, *n* (%)	121 (17.4)	45 (12.0)	0.52 (0.37–0.77)	**0.001**
Chronic disease ≥ 2, *n* (%)	302 (45.8)	316 (74.9)	3.53 (2.70–4.62)	**<0.001**
**Household income, Chinese Yuan**				
Low household income (≤5,000 CNY/month), *n* (%)	130 (19.7)	98 (23.2)	0.93 (0.73–1.19)	0.165
Middle household income (5,000–8,000 CNY/month), *n* (%)	359 (53.1)	222 (53.4)	1.01 (0.84–1.22)	0.565
High household income (>8,000 CNY/month), *n* (%)	171 (25.9)	102 (24.2)	0.91 (0.69–1.21)	0.521
**Quality of life**				
Physical health, (Mean SD)	14.17 ± 2.09	13.72 ± 2.21		**<0.001**
Psychological health, (Mean SD)	13.62 ± 2.18	13.16 ± 2.14		**<0.001**
Social relationships, (Mean SD)	15.49 ± 2.77	15.47 ± 2.80		0.699
Environment, (Mean SD)	14.08 ± 2.71	14.06 ± 2.55		0.909
Total score, (Mean SD)	82.58 ± 9.30	80.04 ± 10.66		**<0.001**

Bold indicates P-values < 0.05.

* Comparison between no depression and depression groups. The data are presented as numbers (%) or mean values (±standard deviation). The Pearson χ^2^ test was used for categorical variables. Student's t-test was used to compare normally distributed variables. Mann-Whitney U tests were used to compare non-normally distributed variables.

In the empty-nest group, variables associated with depression symptoms in the univariate analyses with a *P*-value < 0.20 were included in the multivariate analysis, after adjusting for age, gender, elementary school, middle school, smoking, physical activity, chronic disease ≥ 2, low household income, physical health, psychological health, total score, the results shown that age (aOR, 1.02; 95% CI, 1.00–1.04; *P* = 0.046), chronic disease ≥ 2 (aOR, 3.29; 95% CI, 2.50–4.33; *P* < 0.001) were associated with increased risk of depression symptoms (Table 2), and physical activity (aOR, 0.59; 95% CI, 0.40–0.87; *P* = 0.008), physical health (aOR, 0.93; 95% CI, 0.88–0.99; *P* = 0.026), psychological health (aOR, 0.93; 95% CI, 0.87–0.98; *P* = 0.013), and total score (aOR, 0.97; 95% CI, 0.96–0.99; *P* < 0.001) were associated with decreased risk of depression symptoms (Table 5).

**Table 5:** Multivariable models showing factors associated with GDS-15 ≥ 5 among empty-nest elderly.

	**OR (95% CI)**	P^*^
Age, y (Mean SD)	1.02 (1.00–1.04)	**0.046**
Physical activity	0.59 (0.40–0.87)	**0.008**
Chronic disease ≥ 2	3.29 (2.50–4.33)	**<0.001**
Physical health	0.93 (0.88–0.99)	**0.026**
Psychological health	0.93 (0.87–0.98)	**0.013**
Total score	0.97 (0.96–0.99)	**<0.001**

Bold indicates P-values < 0.05.

* Multivariable adjusted for age, gender, elementary school, middle school, smoking, physical activity, chronic disease ≥ 2, low household income, physical activity, physical health, psychological health, total score.

## Discussion

In this study, we found that the prevalence of depression of no empty-nest elderly group and empty-nest elderly was about 19.8% and 39.0%, respectively, which was line with previous studies (10, 11). We found that age, chronic disease ≥ 2 were associated with increased risk of depression symptoms among empty-nest elderly, and physical activity, physical health, psychological health, total score were associated with decreased risk of depression symptoms. Among empty-nest elderly, the physical and psychological health, and total score of the WHOQOL-BREF scale had the lower scores.

Depression is one of the mental problems that seriously affect human physical and mental health. Previous studies have shown that the prevalence of depression symptoms in the elderly population in China ranges from 13 to 41% (17–20). Many studies had shown that empty-nest elderly had a significantly higher prevalence of depression than the general elderly. According to previous studies, the prevalence of depression symptoms among empty-nest elderly was 30–75% (10, 11, 21, 22). In this study, the prevalence of depression symptoms was 30.1% among all participants, and 39.0% among empty-nest elderly. The different prevalence of depression symptoms might be related to different progress of urbanization, income level, social support and negative or positive coping styles in empty-nest elderly, in rural areas, all resources were relatively limited, social support of the empty-nest elderly was very limited, the elderly lived alone in the countryside, and their peers health also was not very good, many elderly people would rather stay at home than go out, most of them had very little entertainment and interaction. In the other hand, some of the studies were done more than 5 years ago, and different scales were used to assess depression symptoms. Therefore, these different prevalence of depression symptoms were due to time frames, study and analysis methods, and area differences in the study population.

Empty-nest elderly are elderly who live alone or with a spouse, they often lack daily care and emotional support (23). Empty-nest is associated with increased loneliness, which is associated with poor psychological health, and with poor physical heath. Loneliness is a subjective feeling associated with lack or poor relationships. Family members, especially adult children, are important sources of social support for the elderly. Therefore, empty-nest elderly are more likely to be depressed and have a poorer QoL.

Physical disease might limit the ability of the elderly to participate in social activities and leading to increase the risk of loneliness. There was a positive relationship between the number of chronic disease and the risk of depression symptoms, elderly with more chronic diseases are more likely to experience fatigue, anorexia, weight loss, intellectual disability, inattention, and loss of interest. In addition, the co-occurrence of depression and chronic disease can lead to poor prognosis and increased mortality (24–26). In our study, we found elderly with chronic disease ≥ 2 had significantly higher prevalence of depression with those with chronic disease < 2 (36.4% vs. 21.7%, *P* < 0.001), which was more prominent in the empty-nest elderly (51.1% vs. 22.8%, *P* < 0.001), these results are in line with those from previous studies (27).

Many previous studies have shown that physical activity can prevent depression in older adults (28, 29). Physical activity reduces the incidence of depression in older adults, improves self-rated health, and enhances subjective well-being and happiness (29–31). Older adults who engage in regular physical activity (e.g., aerobic exercise, muscular endurance exercise) are much less likely to suffer from depression than those who did not physical activity (31). In our study, we found that physical exercise could reduce the prevalence of depression symptoms among all elderly, it is more significant in the empty-nest elderly. Physical activity can reduce the risk of depression symptoms for several possible reasons. First, older adults who consistently engage in physical activity experience more positive leisure activities and social support, resulting in higher psychological well-being and ultimately less depression (32). Second, physical exercise can have a positive impact on mental health by strengthening social support, such as enhancing friendships and exchanging emotional assistance (33, 34). In addition, physical activity can increase the secretion of antidepressant hormones: physical activity can temporarily change the activity of central norepinephrine, decrease the hypothalamic pituitary-adrenal axis, and increase the secretion of β-endorphin (35). Therefore, continuous physical activity can maintain positive emotions and ultimately prevent negative emotions (36). In particular, depression (such as bereavement) experienced by elderly people living alone is generally chronic rather than acute and can reduce life satisfaction (37). Therefore, community doctors should encourage middle-aged and elderly patients to engage in appropriate physical exercise to reduce the risk of depression symptoms.

The physical health, psychological health, and total score of the WHOQOL-BREF scale had the lower scores in empty-nest elderly. No significant differences in social relationships and environment score were observed between the two groups. Our study suggested that lower QoL, including poorer physical and psychological health, were associated with increased risk of depression symptoms. This result further supported a recent finding (38). Depression is associated with a range of poor outcomes, such as poor health and psychological health; therefore, empty-nest elderly would have lowered QoL. Previous studies had reported similar results (39, 40). Therefore, psychosocial and physical health factors are needed more attention among empty-nest elderly.

This study had some limitations. First, the study was a cross-sectional study, which had some bias, such as nonresponse bias, recall bias (physical activity, income level), which limited the ability to infer causality. Second, GDS-15 was used to assess depression symptoms, which was used to screen for depression, but not to diagnose depression. Third, self-report methods (QoL) were used to collect data, which might overestimate the strength of the relationship between mental health and some variables. Fourth, we did not collect data on medication use, especially mood-improving medication use. Although the study had some limitations, the results of this study could represent the emotional status and QoL of the empty-nest elderly in southwest China, and provide help for the government to formulate corresponding preventive measures.

In conclusion, the prevalence of depression symptoms was 39% among the empty-nest elderly in Chengdu. Age, chronic disease ≥ 2 were the significant risk factors of depression symptoms, and physical activity, physical health, psychological health, total score were found to be significant decreased risk factors of depression symptoms. According to these risk factors, we should take some measures to improve the depression symptoms of empty-nest elderly. Governments and community health doctors should educate people about the benefits of physical activity and encourage older people, especially empty nest elderly, to participate in physical activity. Family members, especially adult children should pay more attention to empty-nest elderly and keep in touch with them as much as possible to improve their physical and psychosocial health.

## Data availability statement

The original contributions presented in the study are included in the article/supplementary material, further inquiries can be directed to the corresponding author/s.

## Ethics statement

The studies involving human participants were reviewed and approved by Ethics Committee of the Second People's Hospital of Chengdu. The patients/participants provided their written informed consent to participate in this study. Written informed consent was obtained from the individual(s) for the publication of any potentially identifiable images or data included in this article.

## Author contributions

LH was responsible for the concept and design of the study, data collection and analysis, and the first draft of the paper and further manuscript. JW was responsible for concept and design of the study, the data analysis, and interpretation. FW was responsible for the data analysis. LZ and YL were responsible for data collection. FX was responsible for overseeing the concept and design of the study. All authors read and approved the final manuscript for publication.

## Funding

This work was funded by the Health and Family Planning Commission of Chengdu (2015009), the Sichuan Medical Association (Q14011), the Chengdu Science and Technology Bureau focuses on research and development support plan (2019-YF09-00097-SN), the Chengdu Medical College Natural Science Foundation (CYZ18-33 and CYZ19-33), the Sichuan Provincial Education Department (17ZA0134), and the Sichuan Medical Association (S18023). The funding body did not participate in designing the study or writing the manuscript. The study protocol had undergone peer-review process by the funding body.

## Conflict of interest

The authors declare that the research was conducted in the absence of any commercial or financial relationships that could be construed as a potential conflict of interest.

## Publisher's note

All claims expressed in this article are solely those of the authors and do not necessarily represent those of their affiliated organizations, or those of the publisher, the editors and the reviewers. Any product that may be evaluated in this article, or claim that may be made by its manufacturer, is not guaranteed or endorsed by the publisher.
